# Controllable Synthesis of Polymeric Micelles by Microfluidic Platforms for Biomedical Applications: A Systematic Review

**DOI:** 10.22037/ijpr.2021.114226.14769

**Published:** 2021

**Authors:** Mahnaz Ahmadi, Saeed Siavashy, Seyed Mohammad Ayyoubzadeh, Rustem Kecili, Fatemeh Ghorbani-Bidkorbeh

**Affiliations:** a *Department of Pharmaceutics, School of Pharmacy, Shahid Beheshti University of Medical Sciences, Tehran, Iran. *; b *Department of Mechanical Engineering, K. N. Toosi University of Technology, Tehran, Iran. *; c *Department of Health Information Management, School of Allied Medical Sciences, Tehran University of Medical Sciences, Tehran, Iran. *; d *Yunus Emre Vocational School of Health Services, Department of Medical Services and Techniques, Anadolu University, Eskişehir, Turkey.*

**Keywords:** Microfluidics, Polymeric micelles, Drug delivery, Nanoparticles, Size distribution

## Abstract

Polymeric micelles (PMs) are one of Nanoscale delivery systems with high stability, loading capacity, and biocompatibility. PMs are nano-sized and spherical particles with a hydrophilic shell and hydrophobic core or reverse depending on their applications. Polymeric micelles could be synthesized by different methods, such as direct dissolution, dialysis method, and lyophilization. Microfluidics is also a relatively modern approach for this purpose, in which chemical reactions are carried out in the microchannels. Compared with conventional preparation methods, the microfluidic technique produces homogeneous polymeric micelles with desirable features, tunable particle size, and relatively high drug loading. These advantages are originated from the ability of microfluidics in precise control over the streamlines of reactants without chaotic turbulence. Although the synthesis of polymeric micelles by the microfluidic platform is advantageous, little or no review has been conducted to provide a clear image of the different PMs preparation by the microfluidic approach. Thus, in this review, the production of the PMs, utilizing microfluidic procedures to enhance their favorable characteristics is investigated. For this purpose, an electronic search is conducted on PubMed, Web of Science, Scopus, and Embase databases for retrieval of relevant papers. Seven papers are included in this systematic review. Preparation of PMs by the microfluidic approach and the effect of different parameters, such as the flow rate ratio, channel dimensions, drug concentration, and organic solvent type on PMs characteristics is obtained from the included papers.

## Introduction

In recent decades, nanosystems have attracted significant attention as a pioneering technology in biomedicine applications. On the one hand, relentless progress made in the nanotechnology field, and on the other hand, the requirements for further improvements in drug delivery accelerate this growing trend in nanomedicine research (1-3). 

The low bioavailability, solubility and efficiency, and high side effects of conventional drugs proved the need for developing more efficient drug delivery systems (4, 5). As a suitable instance, although *in-vitro *studies demonstrate chemotherapeutic drugs are often highly potent, they are generally unable to show sufficient therapeutic efficacy in *in-vivo *research, and this is mostly due to the insufficient retention and accumulation at the target site. At the same time, conventional drugs can cause serious side effects on healthy tissues resulting in undesired problems for patients (6). In order to provide a more efficient drug delivery system with reduced side effects, various types of nanomaterial-based systems (*i.e*., nanocarriers) have been developed (7); including polymeric nanoparticles (NPs) (8), organic-inorganic hybrid nanocarriers (9), liposomes (10), micelles (11), *etc*. NPs have shown good advantages as the therapeutic and diagnostic agent carriers (12). These advantages enable controlling release rate, modifying biodistribution, enhancing the solubility and delivery of poorly aqueous soluble drugs, targeting therapeutic agents to tumors and specific tissues, and transporting therapeutics across the biological barriers, and the ability of transcellular and intracellular delivery drug molecules (13-17). 

Amid the various types of nanocarriers developed in recent years, the self-assembled micellar structures which have been formed by amphiphilic block copolymers have demonstrated many advantages due to their unique features and applications in the field of drug delivery (18). Polymeric micelles (PMs) usually are nano-sized and spherical colloidal particles with a hydrophilic outer shell and hydrophobic inner core or reverse, depending on their applications. While the hydrophilic segments of the polymer molecules form the shell that provides compatibility of the micelles in the aqueous medium, the other portions of the polymer molecules assemble the hydrophobic core of the micelles, which provides the ability of solubilization of the hydrophobic drug molecules into the micelles. The hydrophilic layer plays a vital role as a protective shell of the core and the loaded drugs to inhibit undesirable interactions with the blood and other biological components (19). Furthermore, the biocompatible polymeric segments provide a long circulation period of the entrapment components in the bloodstream because of reduced detection of the micelles by reticuloendothelial systems. In addition, the desired size of PMs (below 150 nm) and their ability of easy surface modification and surface functionalization with ligands make them capable of convenient transcellular and intracellular transfer (20). 

Various amphiphilic copolymers, including graft copolymers, diblock (A-B), and triblock (A-B-A) have been utilized to fabricate polymeric micelles. The most prevalent hydrophilic block in the copolymeric micelles is poly(ethylene glycol) (PEG). Other hydrophilic blocks forming polymers include poly(N-vinyl pyrrolidone) (PVP), chitosan, and poly(N-isopropylacrylamide) (pNIPAAm). There are different types of polymer blocks suggested to utilizing in the formation of the micellar core, including the various polyesters such as poly(L-lactide) (PLA), class of polyethers such as poly(propylene oxide) (PPO), poly(lactide-co-glycolic acid) (PLGA), poly-ƹ-caprolactone (PCL), poly(β-aminoesters), polyamino acids such as poly(L-aspartic acid) (pAsp), poly(L-histidine) (pHis) (21).

Among prevalent self-assembly methods, the bottom-up assembly procedures are well-known as more efficient methods. However, in these manners, driving forces are mainly constituted from weak internalinteractions (22, 23), including electrostatic interaction, hydrogen bond, hydrophobic interaction, Van der Waals force, π−π stacking interaction, and steric forces. Due to the complicated interaction between these factors, it is challenging to stably and precisely control the complex conditions of reactions to fabricate uniform structures. Hence, a reliable complementary procedure is necessary to simultaneously control the conditions of the self-assembly process in both the external environment and internal factors (24). 

Microfluidic technology has been demonstrated as a capable technique to govern both the chemical and physical properties of the reaction medium within a confined environment. The main characteristics of microfluidic systems are the laminar flow regime and dominance of interfacial forces, which provide the ability of precise control over the movement of building blocks and streamlines of reactants without chaotic turbulence (25). Therefore microfluidic systems have been suggested as highly reliable tools to manipulate self-assembly processes over time and space scales, which have been applied to the synthesis of different types of nanoparticles comprising desired micelles (26). The system can produce homogeneous polymeric micelles with desirable features, tunable particle size, and relatively high drug loading compared with conventional methods (27). 

All in all, due to the desirable features of the PMs including the nanoscopic size, biocompatibility, the ability of solubilization hydrophobic drugs in large amounts, and easy surface functionalization simultaneously, which leads to achieving site-specific delivery, PMs have demonstrated promising potential in drug delivery. Although the synthesis of polymeric micelles by microfluidic platforms is advantageous, little or no review has been conducted to provide a clear image of the different PMs preparation by the microfluidic approach. Thus, in this review, the development of PMs preparation utilizing a novel microfluidic approach to enhance their favorable characteristics is discussed. Besides, the effects of various parameters on the PMs characteristics are investigated. Additionally, the microfluidic method is compared with conventional manners to demonstrate the advantages of the microfluidic approach. 

## Experimental

*Method*

An electronic search was performed in four databases, including PubMed, Web of Science, Scopus, and Embase. In the search strategy, keywords related to polymeric micelles were searched at the title. The words related to production were also searched at the title and abstract level. The search was performed without any time restrictions. The search query in the PubMed database is shown in Table 1. In other databases, similar search queries were used. The inclusion and exclusion criteria were as follows. 

*Inclusion Criteria*

1) Matched with the search query

2) The paper includes the preparation of polymeric micelles by the microfluidic method 

3) The paper is in the English language

*Exclusion Criteria*

1) Review papers

2) Papers that the full text was not available

*Screening Process*

All results obtained from four databases were imported to EndNote software. Then, duplicate papers were removed. The title and abstract of the articles were screened by a researcher. 

*Analysis*

Article title, author, year, polymeric micelles composition, influential factors on the characteristics of PMs, and important results were extracted from the included papers and tabulated. This review was carried out based on the Preferred Reporting Items for Systematic Reviews and Meta-Analyses (PRISMA) guidelines (28). 

## Results and Discussion

**Figure 1:**
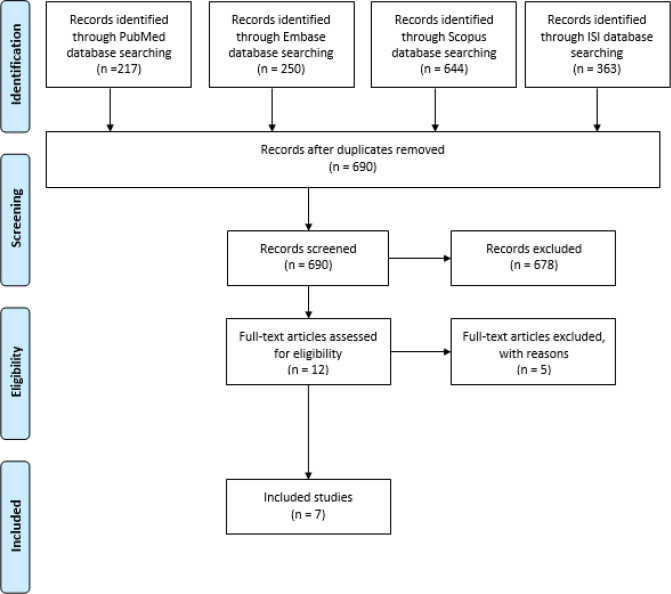
Flow chart of the study selection process based on PRISMA

In this paper, the preparation of polymeric micelles for drug delivery by the microfluidic platform was reviewed. The process of study is illustrated in Figure 1, and the results are represented in Table 2. Seven papers were included in this review. 

*Preparation of polymeric micelles by the microfluidic approach*

**Figure 2:**
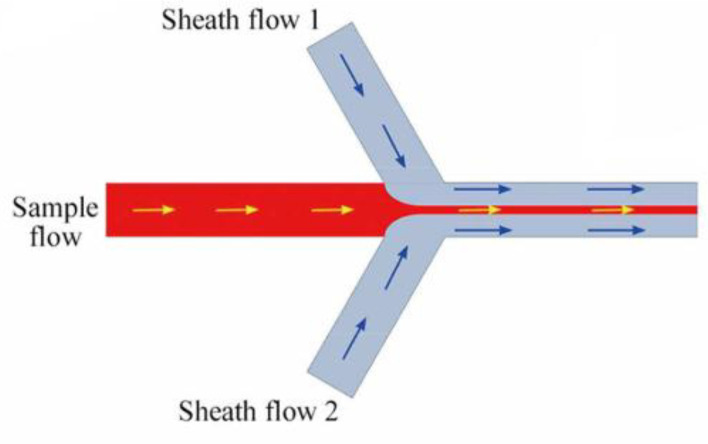
A microfluidic reactor with flow focusing geometry (Adapted with permission from Royal Society of Chemistry (35)).

Lorenzo Capretto *et al. *investigated the production of polymeric micelles in microreactor by microfluidic technique. Microreactor, as shown in Figure 2, consists of three inlets, one main channel, and one outlet. A solution of DMSO and Pluronic polymer was focused hydrodynamically in the central channel through two laterals water flow as non-solvent, and then nanoprecipitation occurred. Pluronic was utilized as a block copolymer because of its well-known features and its safety that approval by the Food and Drug Administration (FDA) for biomedical applications. Comparing the microfluidic system with the batch system showed that the size of the PMs produced using microfluidics was smaller than the PMs of the batch system. Moreover, there was a fluctuation in the mean size of particles when the flow rate was changed in the batch reactor (29). Flow focusing geometry of microfluidics applied in the mentioned study is shown in Figure 2 (35).

**Figure 3:**
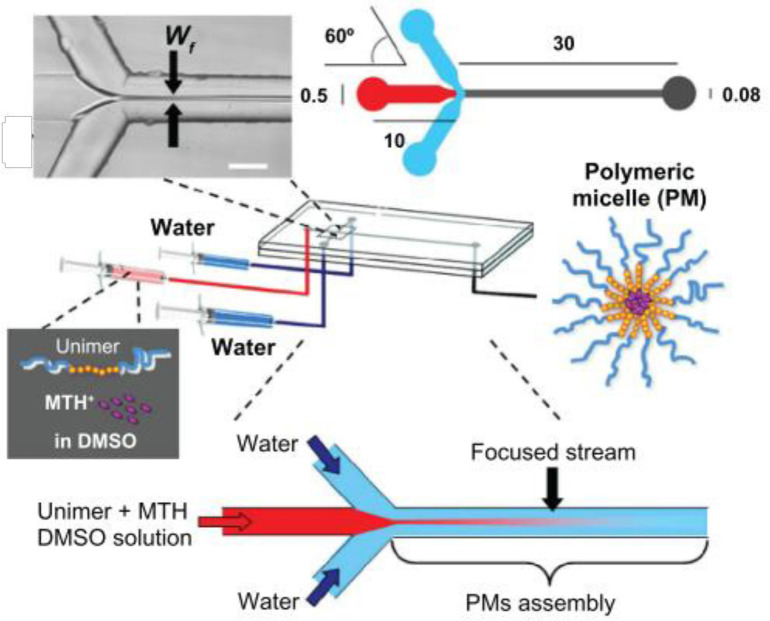
The preparation procedure of polymeric micelles by microfluidics. (Adapted with permission from Dove Medical Press (30)).

In another study by Lorenzo Capretto *et al.*, mithramycin drug was encapsulated into PMs by microfluidic technology. PMs were prepared by a microfluidic reactor consisting of three inlets, one main channel, and a single outlet, as shown in Figure 3. The architecture of channels created a hydrodynamic flow focusing on the main stream. First, polymer and drug were dissolved in DMSO, and the DMSO solution was injected into the microfluidic device. Two side streams of water hydrodynamically focus solution flow in the main channel. Diffusion and solvent exchange occurred. This process triggered the nucleation of PMs. After nucleation and in parallel with growth, the drug was loaded into micelles (30).

Yu Lu, *et al*., produced fluconazole-loaded polymeric micelles composed of amphiphilic diblock copolymers using a microfluidic device. This process was investigated using a co-flow microfluidic device that includes two coaxial capillaries put in each other. Initially, the water phase was injected into the microfluidic device at a fixed flow rate; then organic phase was injected at different flow rates. In these micelles, hydrophobic polycaprolactone (PCL) and hydrophilic polyethylene glycol (PEG) were used in different mole ratios. The drug was dissolved in the organic phase for the preparation of drug-loaded micelles. Besides, the effect of important parameters on the size of polymeric micelles was analyzed (31). 

L. Capretto *et al. *reported the preparation process of polymeric micelles as a drug carrier in order to co-delivery of dexamethasone and ascorbyl-palmitate to *in-vitro* cultured human periodontal ligament mesenchymal stem cells (hPDLSCs). In the study, drugs and polymer were mixed in an appropriate organic solvent, and PMs were produced in the microchannel. Microfluidic reactors fabricated PMs with positive characteristics such as narrow size distribution and high drug loading efficiency. The microfluidic device contained three inlet channels and one mixing channel. All channels had the same width and depth. The solution of polymer and drug in organic solvent flowed into the central channel. The central main stream is hydrodynamically focused when it is confronted with two lateral flows of non-solvent (33). 

Thomas Q. Chastek *et al*., developed a new microfluidic device that integrated the synthesis of block copolymer micelles and measurements through DLS. In this system, the size and size distribution of the self-assembled polymeric micelles were determined by the DLS probe. Figure 1 demonstrates an outline of the microfluidic system that integrates micelles synthesis and in situ particle sizing with dynamic light scattering (34). 

Yuchen Bao and coworkers prepared docetaxel-loaded polymeric micelles based on PLGA-PEG-Mal by microfluidics and dialysis methods. Besides, the physicochemical characteristics and biological effects of PMs were investigated *in-vitro *and *in-vivo.*

The microfluidic approach produced polymeric micelles with a spherical shape, smaller particle size, and a narrower size distribution than the dialysis method. Furthermore, the high drug loading capacity of PMs was obtained by microfluidics. In the bulk methods, control of fluids flow is deficient, and polymers aggregate, so the drug cannot be loaded efficiently. However, in the microfluidics, well-controlled conditions reduce the time of mixing and self-assembly of particles occurs initially when the solvent exchange is finished, so more drug is loaded into micelles. Moreover, PMs prepared by microfluidics showed high tumor accumulation and antitumor efficacy. Arg-Gly-Asp (RGD) was utilized as a targeting agent on the PMs, and the targeting micelles demonstrated higher efficiency in the cancer cells (27). 

*The effect of different parameters on polymeric micelles characteristics*

*Effect of the flow rate ratio*

Volumetric flow rate ratios (R) of the polymer solution to water could be regulated by changing each stream’s flow rate. In the study of Lorenzo Capretto *et al*., the effect of flow rate ratio (R) on the size of the PMs was evaluated by varying R in the range of 0.03 to 0.13. Additionally, the initial concentration of the polymer solution was changed. When R increased, at the polymer concentration of 7.5 × 10^-3^ M, the size of PMs increased from about 100 to 125 nm, and at the polymer concentration of 1.5 × 10^-2^ M increased from 110 nm to 125 nm (29). 

**Figure 4:**
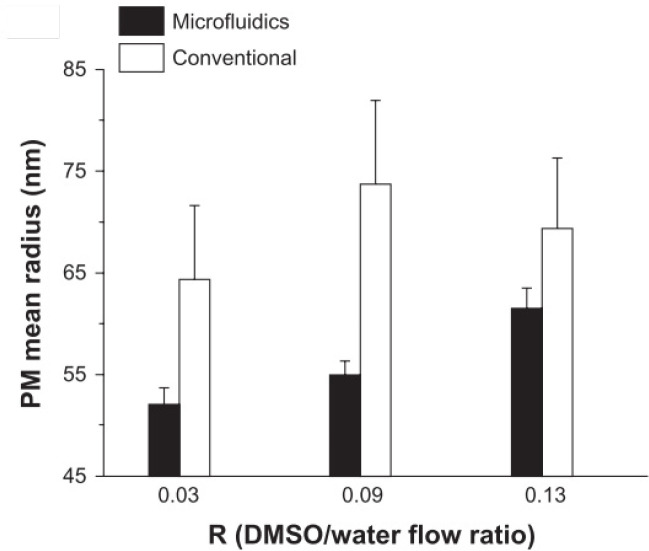
Effects of flow rate ratio (Adapted with permission from Dove Medical Press (30))

Also, in another study by Lorenzo Capretto *et al*., the volumetric flow ratio (R) was considered the first effective parameter. R is correlated to the mixing time between solvent and non-solvent in the production of PMs by microfluidics. The effect of flow rate and comparison of the microfluidic and conventional technique is shown in Figure 4. In the microfluidic method, PMs size demonstrated a linear correlation with R, while there was no clear correlation in the bulk method. Furthermore, microfluidics showed high controllability and reproducibility and more minor standard deviation of the mean radius of PMs compared to the conventional procedure (30). 

The results obtained by L. Capretto *et al. *indicated the notable effect of flow rate ratio (R) on the size of PMs. For example, at a low R, *i.e.* 0.03, the mean diameter of PMs was found 207 ± 28 nm, but at a larger R, *i.e.* 0.13, micelles became very large with the size of 1484 ± 235 nm (33). 

Yuchen Bao, *et al*., proved that the aqueous/organic phase flow rate ratio could influence PMs features. The organic (PLGA-PEG-Mal and docetaxel in acetonitrile) and water phase flow rate ratio optimized in 1:9 ratio and were obtained small particles in this ratio (27). 

*Effect of channel dimensions*

Lorenzo Capretto *et al. *observed that alternation of the channel dimensions in the microfluidic device, particularly the channel width, influences the width of the focused stream and mixing time and so affects PMs features. For example, when the microchannel dimension was decreased, the mean diameter of PMs decreased. Furthermore, smaller microreactor dimensions increase the uniformity of the PMs (29). 

*Effects of polymer concentration*

Another imperative parameter to be considered especially for biomedical applications is the initial polymer concentration. Polymer concentration and critical micellization concentration can be related to PMs stability *in-vivo*. Lorenzo Capretto *et al. *demonstrated that dimensions of PMs slightly increased at high polymer concentration. This small effect was related to increased viscosity of the polymeric solution (30). 

Yuchen Bao, *et al*., demonstrated that polymer concentration is an important factor in the micelle formation process. During the process of micelle formation, the concentration of the polymers in solution is the most important factor. PLGA-PEG polymer showed critical micelle concentration (CMC) 100 times lower than polymeric micelles produced by microfluidics. Moreover, the optimized ratio of polymer to the drug was obtained at a 10:2 weight ratio to reach maximum drug encapsulation and micelle stability (27).

*Effect of drug concentration*

Lorenzo Capretto *et al. *evaluated the effect of mithramycin (MTH) concentration on the size of the micelles in the microfluidic approach. The results showed a slight increase in the size of PMs produced by microfluidics with increasing MTH concentrations from 10 to 55 µM. In contrast MTH concentration demonstrated a significant effect during the conventional method (30). 

In another study by L. Capretto *et al*., the effect of drug concentration was studied. For example, it indicated that when AP concentration was 1.000 mM the size of the PMs is dramatically decreased to a mean diameter of 207 ± 28 nm. This concentration did not cause precipitation problems in a microfluidic device. In addition, more reduction of AP concentration allowed a more reduction in the size of the micelles. Finally, concentrations of Dex and AP were maintained constant in the solution. For instance, based on the results, the optimized concentration of AP was determined by 1.000 mM due to the possibility to reach high loading efficiency for AP into the PMs, and the size of PMs was in the nanoscale range in this concentration (33). 

**Figure 5.**
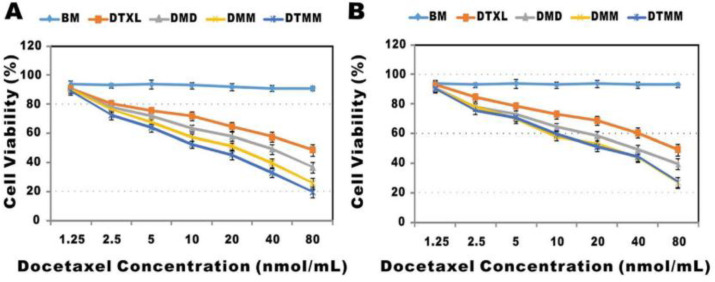
Cytotoxicity of different systems of micelles and docetaxel against two cell lines (A: A549 and B: 3LL cells) (Adapted with permission from Royal Society of Chemistry (27)).

The effect of concentration of docetaxel drug was analyzed by Yuchen Bao, *et al*., and demonstrated the results in Figure 5. Figures displayed cytotoxicity of blank micelles (BM), free docetaxel (DTXL), drug-loaded polymeric micelles prepared by dialysis (DMD), microfluidics (DMM), and targeted polymeric micelles (DTMM). The blank micelles without the drug exhibited almost no toxicity representing the safety of PLGA-PEG-Mal micelles. DMM showed less toxicity compared to DMD with increasing drug concentration. This result indicated that DMM entered into cells more efficiently than DMD and released drugs consistently. Besides, the toxicity of DTMM was lower than DMM due to the targeting effect of RGD (27). 

*Copolymer and organic solvent type*

The type of copolymers and organic solvents are important factors that can influence the mean size of polymeric micelles. Although the organic solvent was evaporated, the solvent may remain in the final product. Using different copolymers in their study, Yu Lu, *et al*., indicated that no significant difference in the micelle size was observed. Furthermore, in this research, THF (Tetrahydrofuran) and acetone were chosen as a solvent because of their low toxicity and good solubility for considered drug and polymer. Results showed that when acetone was used as a solvent, the size of polymeric micelles was larger than using THF, although this difference was slight (31). 

*Aqueous/Organic Phase ratio (Q*_a_ */Q*_o_ *)*

**Figure 6:**
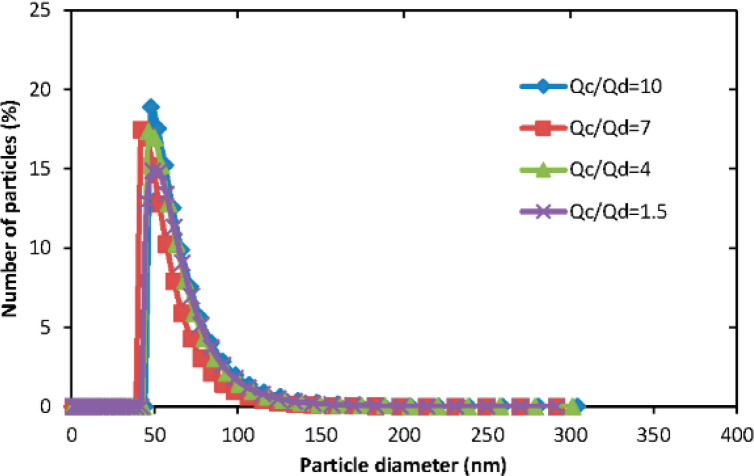
The size distribution of polymeric micelles in different aqueous/organic phase ratio (Adapted with permission from MDPI (31))

Yu Lu, *et al*., investigated the effect of aqueous/organic phase ratio on the size of polymeric micelles. Figure 6 illustrates the particle size distribution of micelles produced from the microfluidic technique. The results showed that there are no significant differences in PMs size in different Q_a_/Q_o_ values in the microfluidic method (31). 

**Table 1:** PubMed search query.

	Search terms
1.	"polymeric micelle"[Title] OR "polymeric micelles"[Title]
2.	(polymeric[Title] AND (micelle[Title] OR micelles[Title]))
3.	1 OR 2
4.	preparation [Title/Abstract] OR production[Title/Abstract]
5.	synthesis[Title/Abstract]
6.	manufacturing[Title/Abstract] OR manufacture[Title/Abstract]
7.	4 OR 5 OR 6
8.	3 And 7

**Table 2:** Included papers regarding the synthesis procedure of polymeric micelles and effective factors on the characteristics of PMs.

Article Title	Author, Year	Polymeric Micelles Composition	Effective Factors on the Characteristics of PMs	Outcomes
Continuous-flow production of polymeric micelles in microreactors: Experimental and computational analysis	Lorenzo Capretto et al. (29) 2011	Pluronic tri-block copolymer	-Flow rate ratio, -Polymer concentration, -Channel dimensions	In the study, polymeric micelles were formed through the nanoprecipitation process in flow-focusing geometry. Organic solutions of the polymer were mixed with a non-solvent, and then the nanoprecipitation process was triggered by the solvent exchange procedure. Afterward, the effect of some important factors such as polymer concentration and flow rate ratio on the size of PMs were investigated. For example, when the flow rate ratio of the polymer solution to water reduced, lower nanoparticle size and narrower size distribution were obtained. As a result, demonstrated that the microfluidic technique presents a platform for the production of polymeric micelles with good reproducibility, high controllability, and uniformity of particle size.
Mithramycin encapsulated in polymeric micelles by microfluidic technology as a novel therapeutic protocol for beta-thalassemia	Lorenzo Capretto et al. (30) 2012	Pluronic F127	-Volumetric flow ratio, -Polymer concentration, -Drug concentration	The study developed a new formulation for mithramycin (as a drug) encapsulated in polymeric micelles (PM-MTH) by microfluidic approach and was compared to conventional methods. Then, in-vitro analysis of the formulation was investigated as a therapeutic procedure. In addition, the effect of flow rate ratio, polymer, and drug concentration on the final PMs was studied. For instance, increasing drug concentration showed a small increase in the size of PMs in the microfluidic approach, but demonstrated a notable effect in the bulk method. The results demonstrated that drugs can be encapsulated into nanoparticles in a controlled manner by microfluidics.
Production of Fluconazole-Loaded Polymeric Micelles Using Membrane and Microfluidic Dispersion Devices	Yu Lu et al. (31) 2016	Copolymer PEG-b-PCL(A), PEG-b-PCL(B) or PEG-b-PCL(C) (With different PEG/PCL mole ratios)	-Copolymer Type -Organic Solvent -Aqueous/organic phase ratio	Fluconazole-loaded polymeric micelles were produced using the co-flow microfluidic device and then outcomes were compared with the membrane-based technique. As a result, the two preparation methods were simple, reproducible, and effective. Also, the effect of copolymer type, organic solvent, and aqueous/organic phase ratio was investigated. For example, demonstrated that the copolymer type used in the study and ratio of phases showed a small effect on the size of the micelles.
Microfluidic reactors for controlled synthesis of polymeric micelles	Lorenzo Capretto et al. (32) 2010	Pluronic F127	-Polymer concentration -Flow rate ratio -Microreactor dimensions	This study demonstrated the relationship between operational parameters and the size of polymeric micelles. PMs were produced in continuous flow microreactors. The dimensions of microreactor had notable effects on the characteristics of PMs produced in microfluidic environment. The preparation process of PMs was based on a nanoprecipitation, with high controllability, reproducibility, and homogeneity of the size of polymeric micelles.
Production of polymeric micelles by microfluidic technology for combined drug delivery: Application to osteogenic differentiation of human periodontal ligament mesenchymal stem cells (hPDLSCs)	L. Capretto et al. (33) 2013	Pluronic F127	-Drugs concentration -Flow rate ratio	The study, reported the production of polymeric micelles by microfluidics, for the co-delivery of dexamethasone (Dex) and ascorbyl-palmitate (AP). Polymeric micelles were investigated in-vitro on the cultured human periodontal ligament mesenchymal stem cells (hPDLSCs). Microfluidic platform produced PMs with high controllability, reproducibility, smaller size, and polydispersity in comparison to the conventional methods. Besides, the effect of drug concentration and flow rate ratio were evaluated. The drug concentration was maintained constant in an optimized amount. In conclusion, microfluidics represented a reproducible manner for the preparation of PMs by controlling the physicochemical properties of nanoparticles essential for biomedical applications.
A microfluidic platform for integrated synthesis and dynamic light scattering measurement of block copolymer micelles	Thomas Q. Chastek et al. (34) 2008	Poly(methyl methacrylate-b-lauryl methacrylate) And Poly(methyl methacrylate-b-octadecyl methacrylate)	-	In the study, a microfluidic device was integrated with dynamic light scattering (DLS) for the synthesis of polymeric micelles and the size measurement of micelles. The DLS probe in the microfluidic device could evaluate the size and aggregation behavior of the micelles. The study illustrated the efficacy of the new synthesis devices for monitoring the behavior of synthesized polymeric micelles.
Engineering docetaxel-loaded micelles for nonsmall cell lung cancer: a comparative study of microfluidic and bulk nanoparticle preparation	Yuchen Bao et al. (27) 2018	PLGA-PEG-Mal-based micelles	-Drug concentration -Polymer concentration -Flow rate ratio	In the study, docetaxel-loaded polymeric micelles were produced by microfluidic techniques and a conventional method (dialysis), and then physicochemical characteristics of PMs were compared. Also, the biological effects of PMs were evaluated in the cell line A549 in-vitro, and also in-vivo in mice. Afterward, the drug and polymer concentration and flow rate ratios were optimized. Compared with the polymeric micelles produced by the dialysis method, the micelles produced by microfluidics illustrated smaller particle size and size distribution, better-sustained release, high drug loading efficiency, and high antitumor efficacy. Results exhibited that microfluidic technology is a promising platform for the preparation of nanoparticles as drug delivery systems.

## Conclusion

In this review, we summarized the preparation process of PMs by the microfluidic approach that has been performed until now. We also investigated the effects of different experimental parameters on the PMs characteristics. The microfluidic-based methods have shown significant advantages. Microfluidics fabricate PMs with desired characteristics such as improved modified size and size distribution and enhanced drug loading efficiency in a precise and thoroughly controllable manner. Recent advances in microfluidics have facilitated the techniques to overcome the challenges involved with the synthesis of PMs by conventional methods. Using the microfluidic platform, it is facilitated to control the chemical reaction conditions by varying the flow rate ratios, channel dimensions, reactant types, and concentrations precisely and conveniently at the same time. Briefly, the integration of microfluidics and drug delivery fields is required to develop more efficient approaches to the fabrication of new PMs with optimal characteristics.
